# Improving Compliance with a Rounding Checklist through Low- and High-technology Interventions: A Quality Improvement Initiative

**DOI:** 10.1097/pq9.0000000000000437

**Published:** 2021-07-28

**Authors:** Leah H. Carr, Michael Padula, John Chuo, Megan Cunningham, John Flibotte, Theresa O’Connor, Beth Thomas, Ursula Nawab

**Affiliations:** From the * Division of Neonatology, Department of Pediatrics, Children’s Hospital of Philadelphia; † Department of Biomedical and Health Informatics, Children’s Hospital of Philadelphia.

## Abstract

**Introduction:**

Checklists aid in ensuring consistency and completeness in medical care delivery. However, using an improvement and safety checklist during rounds was variable in our neonatology intensive care unit (NICU), and completion was not tracked sustainably. This quality improvement (QI) initiative’s primary aim was to increase compliance with checklist completion from 31% to >75% within 1 year.

**Methods:**

A multidisciplinary QI team identified barriers to checklist completion and implemented a human factors-focused low-technology intervention (redesign of a hard-copy checklist) and later a high-technology clinical decision support tool within the electronic health record. The primary outcome measure was percent compliance with the use of the checklist. Process metrics included the duration of checklist completion. Balancing measures included staff perceptions of work burden and question relevance.

**Results:**

Major barriers to checklist utilization were inability to remember, rounding interruptions, and perceived lack of question relevance to patients. Average biweekly checklist compliance improved from 31% before interventions to 80% after interventions. Average checklist completion time decreased from 46 to 11 seconds. Follow-up surveys demonstrated more respondents found questions “completely relevant” (34% pre versus 43% post) but perceived increased work burden (26% pre versus 31% post).

**Conclusions:**

Using QI methodology, human factors-based interventions, and a novel clinical decision support tool, we significantly improved efficiency and checklist compliance and created an automated, sustainable method for monitoring completion and responses. This foundational project provides an infrastructure broadly applicable to QI work in other healthcare settings.

## INTRODUCTION

Following widespread use in aviation and product manufacturing, healthcare providers have employed checklists as a mechanism for improving patient safety and care delivery.^1–6^ Effective checklist implementation has been associated with reductions in postoperative mortality and adverse events,^7,8^ decreased procedural complications,^9–11^ improved communication within intensive care unit (ICU) teams,^12,13^ shortened ICU length of stay,^12^ and increased family engagement in pediatric settings.^14^ However, several factors can limit checklists’ use, including the complexity of patients and their care environments, cultural resistance to checklist use, and lack of perceived relevance of items to patient care.^4,5,15^

In our neonatal ICU (NICU), we care for patients with complex medical and surgical needs. To standardize critical daily aspects of patient care and implement unit-wide quality improvement (QI) and patient safety initiatives, unit leadership developed a checklist for rounding teams. This list, known as the “Clinical Care Questions” (CCQ), has undergone multiple iterations over the last decade. However, variable compliance with completing the list, a lack of sustainable methods for monitoring its use, and an inability to track responses to the questions made it impossible to evaluate its impact on patient care. We primarily aimed to improve the completion of the checklist from 31% of patients before starting any test of change to an average of greater than 75% within a year. Second, we hoped to create a sustainable method of monitoring compliance and question responses.

## METHODS

### Context

The NICU is a 102-bed Level IV unit in a tertiary, university-affiliated teaching hospital. Patients in the NICU are often acutely ill and require coordination with multiple subspecialists. Six teams provide patient care (5 caring for primarily medical issues and one assigned to care of patients with primarily surgical problems). Daily rounds provide an opportunity for the entire patient care team to discuss a patient’s clinical status, plan of care, and allow for communication between the team and family. During rounds, most groups use a workstation on wheels to access the electronic health record (EHR; Epic Systems Corporation, Verona, Wis.) to place care-related orders and look up information pertinent to care planning. The rounding teams consist of attending and fellow physicians, front-line clinicians (FLCs; that is, resident physicians, hospitalist physicians, nurse practitioners, physician assistants), bedside nurses, patient families, and often dieticians, pharmacists, and respiratory therapists. Front-line providers often initiate the CCQ to promote discussion among the whole team. Before this project, the CCQ was displayed as full sentences in black text on a white laminated placard, with questions organized by day of the week. QI leaders placed the placards on the desk platform of computers designated for rounding.

### Design

**Fig. 1.**
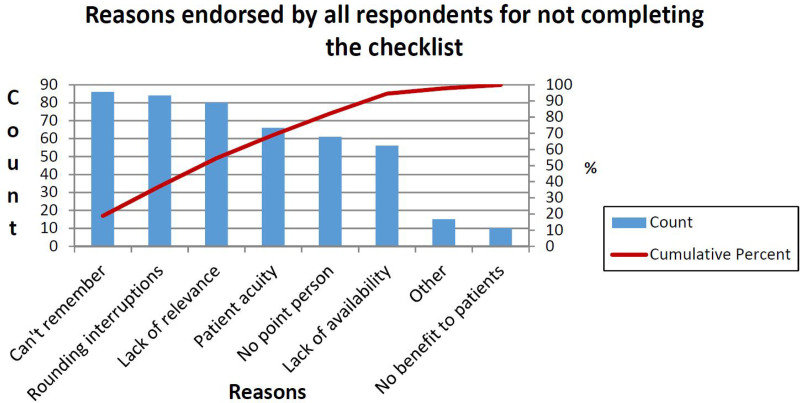
Initial Pareto analysis of provider responses to the initial REDCap survey (n = 202 respondents). Top endorsed reasons for not completing the list were difficulty remembering, rounding interruptions, and a lack of perceived relevance of the questions to patient care.

This QI project used the Institute for Healthcare Improvement’s (IHI) Model for Improvement.^16^ We assembled a multidisciplinary team consisting of physicians, nursing leadership, and a physician assistant who met biweekly throughout the project’s duration. This group assessed barriers to CCQ completion using a REDCap survey^17,18^ sent to all unit bedside nurses, nursing leadership, FLCs, neonatology fellow physicians, and attending neonatologists. The team administered the survey at three time-points: before any intervention, after the unit-wide rollout of our initial intervention, and several months following interventions. We applied a Pareto analysis to the initial survey results to identify and concentrate on the most common barriers to checklist completion. Pareto analysis of the initial survey responses revealed that difficulty remembering, rounding interruptions, and lack of relevance to patient care were the most common barriers to completion (Fig. 1). Though less often-cited, additional contributors included patient acuity, lack of a point person to lead task initiation, lack of availability of the list on the rounding workstation, and a lack of perceived benefit to patients.

### Interventions

**Fig. 2.**
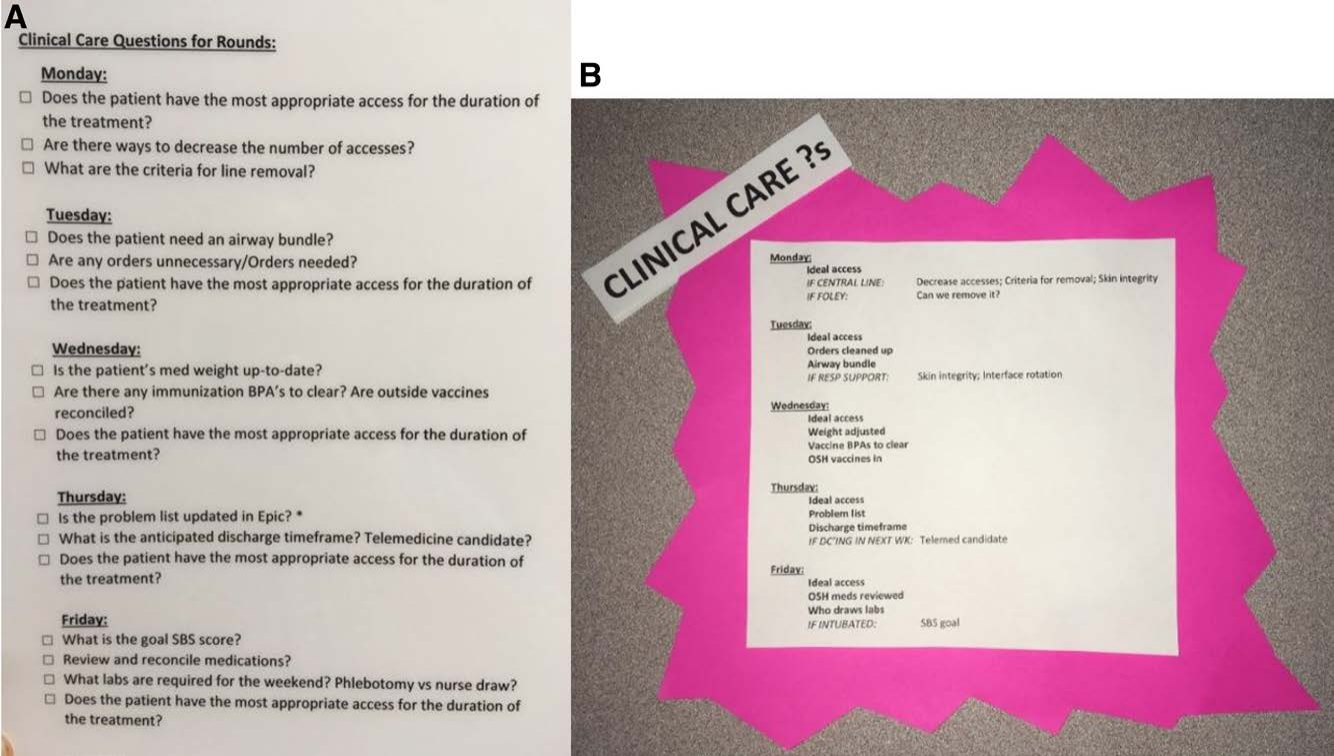
Low-technology checklist modification. Original checklist format (A) compared with the abbreviated version following human factors consultation (B).

To directly address several of the critical barriers to CCQ completion, the QI team designed and implemented 2 major interventions, one designated “low-technology” and one “high-technology.” For the low-technology intervention, we consulted with a human factors engineer to make multiple placard design modifications. Instead of a long-form question list, we used abbreviated keywords displayed on a pink background with a “starburst” appearance. The placard location was moved from the desk platform to the computer screen’s back to fall within the FLCs line of sight. We applied Velcro to make the list easy to remove and replace. This low-technology intervention was rolled out in stages to the six teams in the unit during 5 Plan-Do-Study-Act (PDSA) cycles beginning in November 2018 (Fig. 2).

**Fig. 3.**
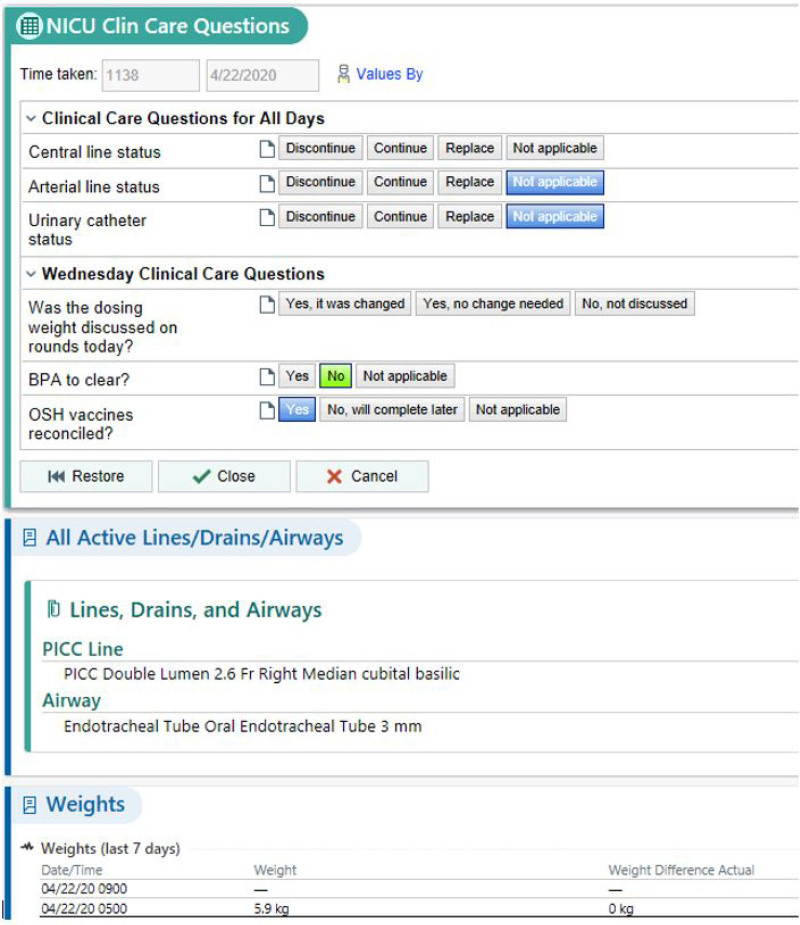
Example of the custom CDS tool from PDSA 6 on a Wednesday when access, weight adjustment, and vaccines are the main points of discussion. As this patient has central access, the tool prompts the provider to provide a plan for the line. As the patient has no arterial access or urinary catheter, these questions are autopopulated as “not applicable.” Also, this patient has no best practice advisory to clear (autopopulated in green), and the information regarding outside hospital vaccine reconciliation has carried over from the previous entry. The tool displays information regarding active lines/drains/airways and specific weight trends to aid with question completion. © 2020 Epic Systems Corporation. Used with permission.

**Fig. 4.**
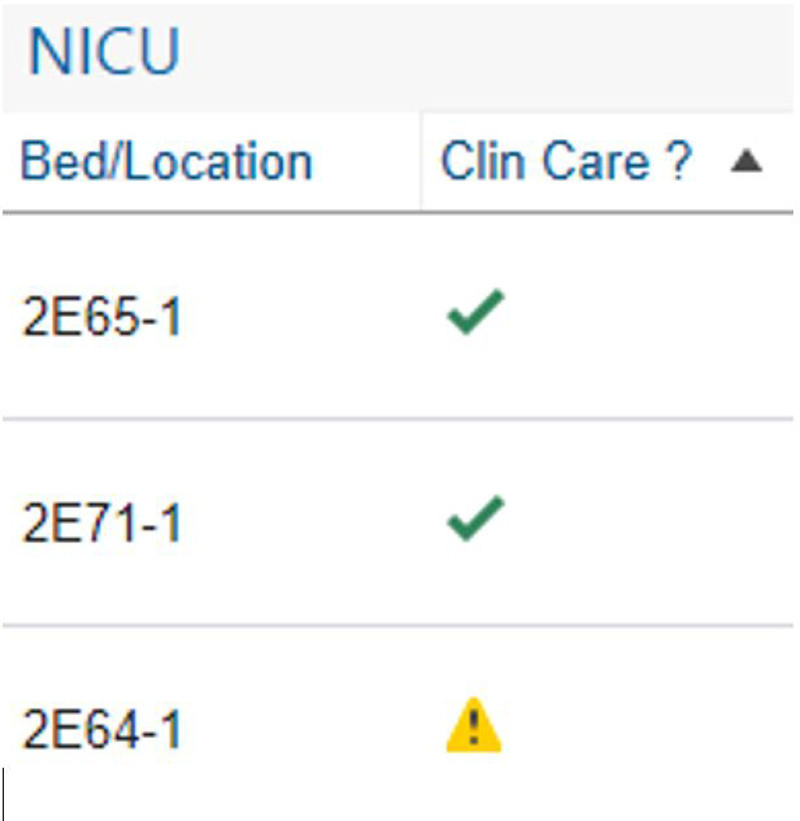
The custom patient list column acts as both an access point and a completion indicator. A caution sign picture indicates a lack of list completion, whereas a green checkmark appears once the provider completes the task. Double-clicking on the icon pictured will bring a provider to the task. © 2020 Epic Systems Corporation. Used with permission.

A physician member of the study team (L.H.C.) designed and programmed the high-technology intervention, which consisted of a clinical decision support (CDS) tool within the EHR (Fig. 3). We implemented this tool (PDSA 6) across the entire unit (all 6 teams) in October 2019, eight months after our initial low-technology intervention, which it replaced. The CDS tool utilized rule-based logic to present only relevant daily questions to care teams based on patient status and day of the week. Also, it autopopulated with appropriate question responses whenever possible (eg, if a patient did not have central access, the tool would autopopulate with “not applicable” when the team discussed a central line plan). The tool was accessible through three points that were part of the FLC workflow: from the patient list, from a link embedded in a daily rounding report, and from a tab on the side of the provider screen. An icon on providers’ patient lists would change from a caution sign to a checkmark to indicate that the task had been completed (Fig. 4). Ten months after PDSA 6, we updated the CDS tool to reflect current QI and patient safety initiatives in the unit. We modified it to make information irrelevant to specific patients not visible (PDSA 7).

In addition to these interventions, staff and nursing leaders announced reminders about the CCQ at daily, unit-wide, and nursing morning huddles starting in February 2019. Throughout the project, team members provided feedback on compliance with unit staff during monthly conferences and electronic kudos through the hospital’s online compliment system.

### Study of the Interventions

We tracked this process from August 2018 through September 2020. We designated the first 3.5 months of data collection (August 2018 through mid-November 2018) as the preintervention period. We designated the postintervention period as a beginning in mid-November 2018 with PDSA 1 through follow-up to mid-September 2020, roughly 22 months of monitoring.

### Measures

Our primary measure was a process measure, which was percent compliance with the use of the CCQ. From August 2018 through March 2020, an unannounced observer who was present for rounds recorded CCQ completion status in a REDCap database. For the preintervention period, we assessed compliance as recorded by the observer. After introducing the CDS tool in October 2019, we assessed compliance through an automated dashboard that extracted provider responses from the EHR. From October 2019 through March 2020, we validated compliance noted in the EHR with data from the unannounced observer. A process measure was the duration of checklist completion. Throughout the project, including before and after both the low- and high-technology interventions, the observer noted the time needed to complete the CCQ and recorded this in a REDCap database. Due to the COVID-19 pandemic, unannounced observers were no longer included in rounds starting in March 2020; therefore, we could not measure the duration of checklist completion after March 2020. Also, we included the number of daily cases assessed as a process measure. Balancing measures included provider’s perceptions of question relevance to patient care and burden related to list-completion reported in anonymous REDCap surveys as discussed earlier. We assessed question relevance and burden with items using Likert-type scales with response options “completely relevant,” “somewhat relevant,” “neutral,” “somewhat irrelevant,” or “completely irrelevant” and “not at all burdensome,” “somewhat burdensome,” or “very burdensome.” Unit team members completed surveys before starting interventions, following the low-technology intervention, and following the high-technology intervention.

### Statistical Analyses

The team analyzed data in 2-week increments and recorded and plotted the primary outcome on a P-type statistical process control (SPC) chart. We assessed secondary process measures using X-bar and S SPC charts. Before any intervention, the initial 8 points were used to determine a centerline in the P-type SPC chart and carried this forward until we noted special cause variation. For the X-bar and S SPC charts assessing duration, we used the initial seven points to determine a centerline, accounting for one week when the unannounced observer did not record completion time. We defined special cause variation in line with prior reports as 6 consecutive points increasing or decreasing, 8 consecutive points above or below the centerline, 2 out of 3 consecutive points near the control limits, a point noted outside the control limits, and 15 consecutive points within one SD of the centerline.^19^ The team assessed balancing measures using descriptive statistics to analyze responses to surveys.

### Ethical Considerations

Our Institutional Review Board evaluated this QI initiative and determined that it did not meet human subjects research criteria. We wrote this publication according to SQUIRE 2.0 guidelines.^20^

## RESULTS

### Primary Outcome

**Fig. 5.**
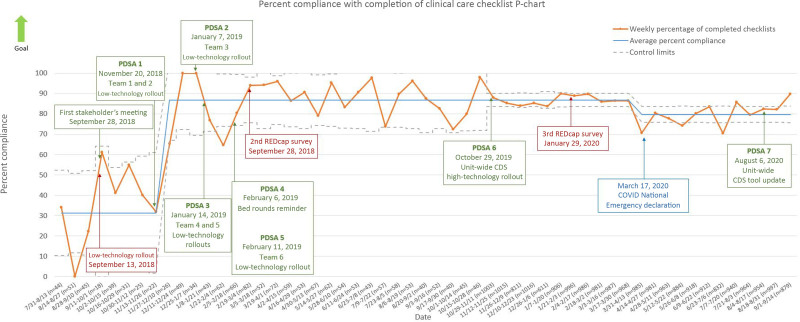
P statistical control chart indicating a significant improvement in compliance. We calculated an initial centerline based on the first 8 points, which shifted at the initial point of special cause variation. We note our centerline at 31%, with improvement to the current centerline of 80%.

There was a significant improvement in the completion of the checklist. Before PDSA cycle 1, the mean compliance was 31%, with an increase in improvement to 87%. Compliance later decreased to 80% following the declaration of the COVID-19 pandemic as a United States national emergency. After PDSA 6, there was a decrease in variability of compliance and marked narrowing of control limits because of a substantial increase in the number of observations reported (Fig. 5).

### Secondary Process Measures

**Fig. 6.**
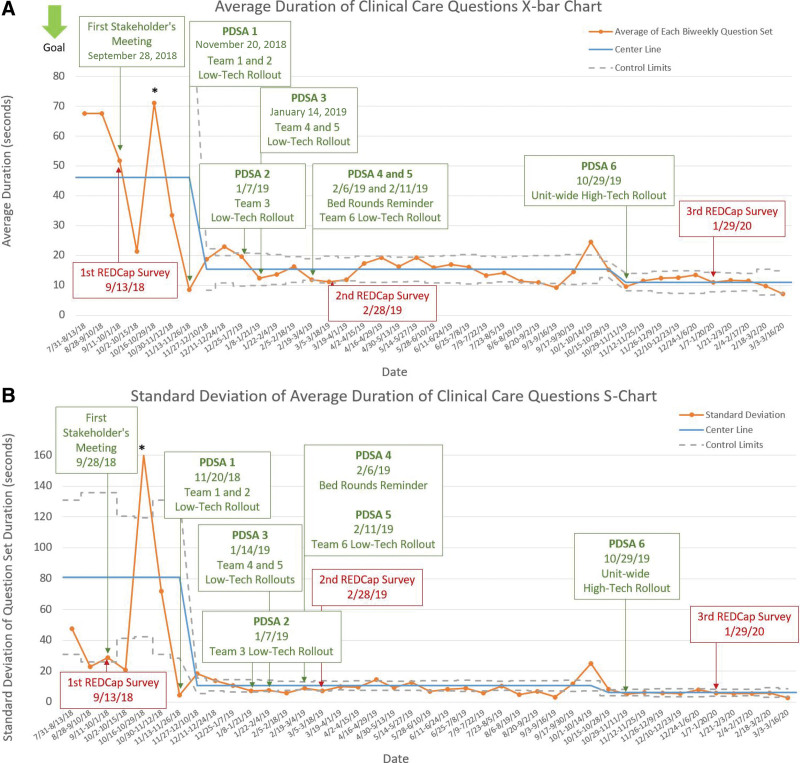
A and B, X-bar and S statistical process charts indicate a significant decrease in list completion time. We noted an initial centerline based upon the first 7 points with a shift at the initial sign of special cause variation at the low-technology release time. Centerline mean completion time was 46 seconds with a SD of 81 seconds, which decreased to 11 seconds with a SD of 9 seconds. The final shift coincided as the high-technology test of change was released to the unit. *Likely data entry error noted.

Completion time decreased significantly throughout the project (Fig. 6a and 6b). Before the first PDSA cycle, completion took an average of 46 seconds, which decreased to 11 seconds. Our average daily observations increased from 4.6 during the preintervention period and before the high-technology intervention (PDSA cycles 1–5) to 96 with the electronic tool, reflecting that the automated tool allows for evaluation of the entire unit census.

### Balancing Measures

Throughout the 3 survey periods, the proportion of providers endorsing the CCQ as “somewhat” or “completely relevant” remained stable (88%, 83%, and 87%), and the proportion of providers endorsing them as “completely relevant*”* increased from 34% initially (preintervention) to 42% on the secondary survey (post low-technology intervention), and 43% on the final survey following the high-technology intervention (n = 165 on the initial survey, n = 125 on the secondary survey, n = 117 on the final survey). Perceptions of burden increased throughout the project, with 26% of respondents initially reporting some degree of burden, 37% on the secondary survey, which then decreased to 31% on the final survey (n = 160 on the initial survey, n = 120 on the secondary survey, n = 119 on the final survey).

### Barriers to Completion

A Pareto analysis of repeat surveys showed a shift in the most commonly cited reasons for not completing the list. Following the low-technology intervention, the top noted reasons were rounding interruptions, difficulty remembering, patient acuity, and lack of relevance, respectively. After the high-technology intervention, the noted reasons shifted again, with rounding interruptions remaining the top reason, followed by patient acuity, and difficulty remembering became the third most-noted reason, lacking perceived relevance in the fourth most-cited spot.

## DISCUSSION

We demonstrated significant improvement in compliance with a quality and patient safety rounding tool in a large NICU through an initial low-technology intervention. We maintained compliance and reduced variation in practice through the use of a high-technology intervention. We also created a sustainable method for monitoring compliance and recording responses to the rounding tool’s questions. Our interventions also yielded a product associated with reduced time to checklist completion and improved modifiable barriers to completion.

Effective implementation of checklists is associated with improvements in many aspects of healthcare,^7–14^ but implementation can be challenging, especially in practice locations of high volume and acuity.^4,5,15^ Human factors, the study of how humans function in a system, is an essential component of a successful healthcare improvement strategy.^21^ By considering how our providers worked in the initial state and addressing the most commonly reported causes for not completing the list (eg, difficulty remembering, perceived lack of question relevance to patient care, etc.), we were able to create meaningful interventions. The low-technology modifications to the placard resulted in substantial improvement in compliance.

Transition to a high-technology CDS tool is associated with its significant challenges.^22^ However, we continued to maintain improved compliance after this intervention and saw a reduction in compliance variability. There was a slight decrease in compliance concurrent with the COVID-19 national emergency declaration, which likely impacted this project’s prioritization as the unit rapidly designed and implemented new safety policies. Despite this change, we observed sustained compliance since March 2020, which is currently increasing. We believe that our CDS tool was successful because it drew from a foundation of culture change established by the low-technology placard. As noted, we saw a transition in our providers’ endorsement of difficulty remembering to complete the list and improvements in perceived question relevance before our high-technology intervention. This change, coupled with consideration of human factors, likely aided in success with the execution of our high-technology intervention.^23^ We suspect that several features of the CDS facilitated its completion. These features include that the list was readily available through parts of the EHR regularly accessed by our units’ providers (patient list column, a hyperlink in a rounding report, and sidebar tab). A change in the patient-list icon appearance after the list’s completion served as a meaningful reminder to complete the task. Also, sorting the questions using patient-specific information and rule-based logic to make the appearance of only appropriate daily questions aided in the ease of completion by excluding irrelevant information. We noted improvement in perceptions of “complete relevance” of patient care questions throughout the project. There was also an increase in burden following the low-technology intervention, which can likely be attributed to the task’s improved completion. As we developed the high-technology intervention with workload reduction in mind, we were pleased with the decrease in burden noted following its introduction.

Our project has several limitations. With little baseline data and substantial variability before the start of the first stakeholder’s meeting, it is possible that we did not have an accurate understanding of the initial state. However, given our baseline values, our SPC charts’ centerlines likely represent an underestimation of change. This project was also completed in a single, large, academic NICU, using an EHR with CDS tools that could be modified by our team. We recognize that these center-specific characteristics, such as informatics expertise, might limit generalizability. There is also the possibility that the presence of our unannounced observer may have influenced provider rounding behavior. We also recognize the inherent limitations associated with the use of self-reported balancing measures. Finally, although a barrier to completing the CCQ cited by providers was rounding interruptions, we could not address this specific barrier with our interventions.

Using QI methodology, our group has created a foundational infrastructure that healthcare providers could use as a framework for future improvement work in various settings. Although this project was mostly process-focused, behavior change, particularly in large, complex settings, is difficult. We look forward to future modifications of this checklist and plan to evaluate the impact on patient outcomes specific to the questions.

## CONCLUSIONS

Through sequential low- and high-technology tests of change focusing on human factors, we improved provider compliance with a rounding checklist in the NICU.

## DISCLOSURE

Dr. Carr is a partial owner and Managing Editor of the medical news and educational website, www.2minutemedicine.com, unrelated to this work. The other authors have no financial interest to declare in relation to the content of this article.

## ACKNOWLEDGMENTS

The authors of this work would like to thank Matt Devine for his assistance in data extraction and presentation, Dr. Michael Posencheg for his QI insights and, Drs. Anthony Luberti and Eric Shelov for their clinical informatics expertise and willingness to troubleshoot during the EHR build, Kristin McNaughton, MHS, for editing.
